# Is there an association between low dose aspirin and anemia (without overt bleeding)?: narrative review

**DOI:** 10.1186/1471-2318-10-71

**Published:** 2010-09-29

**Authors:** Helen Gaskell, Sheena Derry, R Andrew Moore

**Affiliations:** 1 Department of Clinical Geratology, John Radcliffe Hospital, Headington, Oxford, OX3 9DU, UK; 2 Pain Research, Nuffield Department of Anaesthetics, University of Oxford, Churchill Hospital, Oxford, OX3 7LJ, UK

## Abstract

**Background:**

Overt bleeding associated with low dose aspirin (LDA) is well-recognized, little attention is given to the possibility of association between LDA and occult bleeding, although this is known to occur in healthy volunteers. LDA is used increasingly in primary and secondary prevention of a number of medical conditions, many of which are common in older people, as is anemia. Anemia in older people is associated with adverse outcomes including disability, morbidity and mortality. The purpose of this study was to review the evidence that LDA might cause anemia without overt bleeding.

**Methods:**

An extensive narrative review was carried out. Electronic searching (including database links) and reference lists of reports were used to identify studies reporting on use of aspirin ≤325 mg/day and anemia or change in hemoglobin (Hb) without overt bleeding. Data were extracted from reports of trials, adverse drug reactions (ADRs) and prevalence studies of adults aged ≥18 years, published since 1980.

**Results:**

There are few relevant data, with considerable heterogeneity among trial designs, duration, and patient characteristics in studies of LDA. In five randomised trials (n = 5879) in (mostly secondary) prevention, the majority of patients were men without peptic ulcer disease aged 50-70 years and no consistent association between LDA and change in Hb was found. In two smaller studies (n = 609) of primary prevention in healthy patients aged ≥70 years, there was a small but statistically significant fall in Hb with LDA. Observational studies, and data from trials in which use of LDA was not a primary focus of the study, were inconclusive.

**Conclusions:**

It is not clear whether there is an association between LDA and anemia in the absence of overt bleeding, but there may be an association between LDA and fall in Hb in (a subset of) older patients. The available evidence has significant limitations, which are discussed; studies including more older patients, and publication of individual patient data, would help clarify this important matter.

## Background

Low dose aspirin (LDA) is taken by an increasing number of people for prophylaxis in a widening variety of medical conditions, including cardio- and cerebrovascular disease, dementia, and some malignancies. Many of these conditions are common, particularly in older people, and changes in demographics (with increasing life expectancy and higher numbers of older people) mean that there is potential for many more older people to take LDA. Improved chance of survival for patients with some of these conditions (after myocardial infarction, for instance) contribute to increased life expectancy, and indeed, may result partly from the use of LDA as prophylaxis. The place of LDA is not yet fully identified, particularly in primary prophylaxis in some conditions. Furthermore, evidence obtained from studies on younger people may not be applicable to older patients.

Although there have been many studies reporting on overt gastrointestinal bleeding as a primary or secondary outcome of interest [1,2], little attention has been given to the possibility that LDA use may be complicated by occult bleeding, possibly leading to anemia. There is some evidence from experimental studies that LDA is associated with increased occult fecal blood loss in healthy young volunteers [3].

Anemia is common in older people [4,5]. It may be asymptomatic, but it is associated with increases in mortality [6-9], morbidity (in a number of conditions) [4], falls [10,11] and disability [12-14], as well as deterioration in cognitive function [15-17]. Even mildly low Hb levels (above the WHO criteria for anemia, Hb < 130 g/L in men and <120 g/L in women [18]) have been found to be associated with significant decline in quality of life in older people [19].

There are other implications beyond the wellbeing of individuals, particularly for costs of health care for an ageing population. Because of the importance of anemia and the uncertainty as to whether taking LDA has a clinically significant effect on Hb (other than in the context of overt bleeding), we carried out a review to examine whether there is any evidence of an association between use of LDA and anemia.

## Methods

We searched for evidence from systematic reviews, randomised trials, observational studies of clinical practice and epidemiological data in several areas:

1. Evidence of changes in Hb during treatment with LDA in randomized trials, where LDA was used as either primary treatment or as a comparator drug. In trials where LDA was used both alone and in combination with another antiplatelet drug (or with an anticoagulant), data were extracted from the trial arm in which LDA alone was used.

2. Evidence from randomized trials in which change in Hb or anemia was reported as an adverse event (ADR) occurring in patients treated with LDA (or evidence that no such ADR had occurred).

3. Evidence from prevalence studies, including data on LDA prescribing and prevalence of anemia (and/or Hb) in different clinical situations.

We considered data from studies on adults in which LDA was used either in primary or secondary prevention, with a maximum dose of 325 mg/day. Data on overt/acute bleeding episodes were excluded. We noted authors' definitions of anemia where this differed from the WHO criteria. We also noted data on co-morbidities and exclusions from studies.

Several different search strategies were used to find full publications of studies relating to these outcomes. These were predominantly free-text searches of PubMed, EMBASE (accessed through the Ovid interface), and the Cochrane Library, to November 2009, including, but not limited to, aspirin, acetylsalicylic, an(a)emia, h(a)emoglobin, h(a)ematocrit, prophylaxis, adverse. We also carried out searches using additional search terms such as cardiovascular, dementia, cancer. A specimen search strategy is shown (in Additional file 1: Specimen search strategy). The sensitivity of electronic databases for observational studies is known not to be high [20,21], so bibliographies of papers, reviews and "linked articles" in PubMed were extensively searched for references to clinical and other potentially relevant studies.

Any study that might have contributed was obtained in full and read. For inclusion, the only criterion was that of full publication; abstracts or posters were not accepted. Formal quality scoring of included studies was not considered feasible because of the likely mix of systematic reviews, randomised trials, and observational studies. Because of the problems in searching for data on events that were often not considered as primary, secondary or even adverse event outcomes, and the enormous number of reports that could potentially contain relevant data, we did not feel able to call this review systematic. We consider it to be an extensive narrative review. There was no pooling of data; no statistical methods were planned, and none used.

## Results

### 1. Clinical trials

There were seven randomised trials (six double blind and placebo controlled) in which LDA was used at doses of 50 mg to 324 mg daily and Hb or related hematological data were reported as primary or secondary outcomes [22-28]. Study details and results are shown in Additional file 2 (Additional file 2: Trial data: LDA as either primary treatment or as comparator drug). Relevant data were available from 6488 patients. Trial duration varied between 12 weeks and six years although the nature of some of the trials was such that there were few data available from patients at longer durations of treatment because of withdrawals.

The trials fell into two groups. In five studies of LDA in (mostly secondary) prevention of cardio- and/or cerebrovascular disease [22,23,25-27] the majority of the 5879 patients were men aged 50-70 years old, and mean baseline Hb was between 133 and 151 g/L [25-27]. Reported co-morbidities were similar and of the sort that might be expected (such as hypertension), and half were current or ex-smokers. Peptic ulcer disease and severe co-morbid illness were commonly excluded. In two studies [22,23] falls in Hb or Hct after baseline were reported in patients taking LDA and in those taking placebo. There was no significant difference between results from the treatment and placebo arms in either study. Mean Hb in both the LDA and placebo arms did not change in the first year after baseline, but fell in the second year of a large study [26,29,30]. Unfortunately, there is no report of the statistical significance of either changes in mean Hb or Hct from baseline to one or two years in each group, or of differences between the mean Hb or Hct in the two groups at a particular time. However, the samples are large, and it is possible that the fall in Hb seen in both groups over the second year might be statistically significant even though it is small. A significant *rise *in Hb at 3 months after baseline was reported from a small study in which LDA was used as a comparator drug (no placebo arm), with a similar value at 4 years [27]. Very small *rises *in Hb (not statistically significant) were also reported in another study [25,31], with no significant difference between results from treatment and placebo arms; however, the magnitude of the standard errors suggest that the underlying distributions of the data may have been skewed.

In two smaller studies [24,28], enteric coated (ec) aspirin 100 mg was prescribed for primary prevention of cardio- or cerebrovascular disease in 609 patients aged 70 or older, with similar numbers of men and women. Patients included in these two studies had similar mean baseline Hb (142 and 140 g/L respectively), were in good general health, and 24% [24] and 44% [28] were current or ex-smokers. In both of these studies there was a statistically significant fall in Hb at one year compared with baseline in the LDA arms of the trials, but not in the placebo groups; there were also statistically significant differences in Hb between LDA and placebo groups at one year in both studies. One study [24] reported the percentage of patients in whom Hb fell by 10 g/L or more, and here too there was a statistically significant difference between the LDA and placebo groups, although there was no significant difference between the two groups with respect to the percentage of patients with any fall in Hb over one year.

### 2. Anemia or change in Hb reported as an ADR

Relevant adverse event data (fall in Hb or Hct, or anemia) were available from several studies [32-38] in which LDA at doses of 75-325 mg daily was used. There was considerable heterogeneity among the study designs. The timing of adverse events was not reported and where anemia was reported, the criteria for diagnosis were not stated explicitly. Study details and results are shown in Additional file 3 (Additional file 3: ADR data: LDA as either primary treatment or as comparator drug).

The prevalence of relevant adverse events ranged between 0% and 4.4%. In one study [37] anemia (defined using a probable criterion of only Hb <90 g/L [39]) occurred less frequently in patients taking LDA at a dose of less than 162 mg daily than in those taking LDA 162-325 mg daily, 70 patients (3.0%) versus 97 patients (4.4%) (p = 0.011).

### 3. Prevalence studies

We found data on prevalence of LDA prescribing and anemia (or Hb levels) in three studies in community settings [40-42], noted as patient characteristics in three studies of treatment of cardiovascular disease in older patients [43-45], and in four studies in secondary care in patients considered for investigation of anemia [46-49]. The majority of the data were from older patients. Study details and results are shown in Additional file 4 (Additional file 4: Prevalence data: LDA use in various settings).

#### Prevalence studies- community settings

The prevalence of LDA prescription in a primary care population and the mean change in Hb over time (in the absence of overt bleeding) were observed [40]. There was a statistically significant fall from baseline in mean Hb in the men and a *rise *(not statistically significant) in mean Hb in the women over a mean period of 25 months. Related data from small subgroups of patients were also reported, including the observation that mean Hb fell by over 10% in 15% of older patients taking LDA. Cross sectional data from two cohort studies [41,42] of similar characteristics showed no statistically significant independent effect of LDA on Hb [41] and a slightly *higher *Hb (and lower prevalence of anemia as defined by WHO criteria) in LDA users than in non-users [42].

#### Prevalence studies - data from patient characteristics in three studies of treatment of cardiovascular disease

Data on aspirin use (probably LDA) and Hb are reported from sub-analyses in studies on the outcomes of treatments of chronic stable angina [45] and chronic heart failure (CHF) [43]. One study found no difference in the prevalence of aspirin (probably LDA) prescription in anemic and non-anemic patients, anemia being defined by WHO criteria [45], but in another study [43] mean Hb was slightly lower in aspirin users compared with non-users (the difference was statistically significant).

The relationship between prior aspirin use and outcomes was examined in a very large study of almost 119,000 patients admitted to hospital with acute myocardial infarction [44]. Aspirin use before admission was associated with a small but statistically significant *higher *mean Hb; dose and duration of aspirin use were not known, but it was likely to have been LDA for most patients.

#### Prevalence studies- data from secondary care

Several studies of patients referred to secondary care for assessment of iron deficiency anemia (IDA) have reported aspirin use as a patient characteristic [46-48]; unfortunately none of these studies had assessment of a relationship between aspirin consumption and Hb/iron status as a primary outcome. Aspirin was taken by 25-29% of patients who had no symptoms that suggested a cause for IDA [47,48]. In an audit of patients referred with IDA, 24% were taking aspirin, but in a subgroup (53%) in which no cause of IDA was found, 41% were taking aspirin, while the prevalence of regular aspirin prescription among patients of similar age in the same catchment area as those referred for investigation of IDA was 11% [46]. Although these are very small studies with limited information reported on indications for aspirin prescription, aspirin dosage, duration of consumption, co-morbidities and other possibly relevant medication (such as NSAIDs), it is likely that the patients in all these studies took aspirin for prophylaxis rather than as analgesics.

In a more recent prospective study [49] of investigation of IDA in patients admitted as inpatients to general medical services (apart from admissions for acute GI bleeds), mean Hb and prevalence of LDA prescription were reported with other related data. There was no information on prevalence of LDA prescribing in the catchment area from which the hospital received its acute admissions.

## Discussion

The most striking observation is that there is so little relevant information on possible covert effects on Hb with such a commonly prescribed drug as aspirin, despite its well-known propensity to cause significant overt gastrointestinal bleeds in some patients. We are aware of the difficulties in identifying all relevant data, (particularly from studies other than trials of LDA in which change in Hb was a primary outcome) but, given the small amount of such trial data available, feel that it is appropriate to present the additional data that we have found, despite their potential limitations. The relevant available data (from a wide range of types of study and outcomes) show no consistent association between LDA and anemia. While some studies indicated that Hb levels were reduced with LDA, others showed no change, or even suggested a higher Hb level. Anemia, however defined, was not consistently associated with aspirin use. We believe that there are a number of factors relevant both to the lack of data, and to the interpretation of what data are available.

The limited trial evidence shows that there is either no effect of LDA on mean Hb, or a very small effect but with considerable variability compared to the effect size. However the trial data should be interpreted with caution; there is considerable clinical heterogeneity among the studies (indication for LDA, dose of LDA, age of participants, duration of trial, data collected, exclusions, and co-morbidities), making it difficult to draw any firm conclusions on the effect of LDA on Hb in the absence of overt bleeding. The majority of trial data comes from men aged 50-70 years with cardio- and/or cerebrovascular disease, including a high proportion of smokers. There are very small amounts of trial data on LDA and Hb in primary prevention in healthy older patients, and it is not reassuring to find that the trial evidence available suggests that in these patients LDA is associated with a fall in Hb, at least when taken for one year [24,28]. We lack information on other groups, in particular on secondary prevention in older people, whether fit or frail. Conditions where LDA prescription may be appropriate are common in older people and many clinicians will recognize the common scenario of having to decide on prescribing LDA for secondary prevention in a mildly anemic elderly patient.

Exclusions, withdrawals from trials, and compliance issues may have had an important impact on the results. The trial data largely precede the widespread use of proton pump inhibitors (PPIs) or Helicobacter pylori eradication therapy; patients with a history of peptic ulcer were excluded from trials of LDA, and the results of these trials may not be readily applicable to patients who take PPI drugs with LDA. There is little formal reporting of reasons for withdrawal from these trials (except where withdrawal occurred because a pre-determined end-point was reached, such as a cardio- or cerebrovascular event in trials where LDA was used for secondary prevention), but aspirin intolerance associated with upper gastro-intestinal symptoms is common. If such symptoms are associated in some patients with occult bleeding sufficient to cause a fall in Hb, there is potential for bias in trial results if affected participants withdraw from a trial. There might be similar bias with (lack of) compliance with LDA [50]. A large Scottish study of patients (mostly aged 60 or more) showed that compliance at a year was less than 50% [51].

Many patients taking LDA have co-morbidities which themselves may be relevant to the development of anemia (chronic heart failure would be one example). Such co-morbidities, and drugs used to treat them, make it more difficult to identify effects on Hb specific to LDA. In particular, not all studies indicated whether patients taking NSAIDs were excluded, and possible effects of even short term or intermittent NSAID use in a longer-term study are difficult to evaluate.

In studies where Hb is recorded during acute illness, the timing of blood sampling can be critical because Hb can vary during acute illness [27,52]. This phenomenon is relevant to trials on LDA in secondary prevention where baseline Hb data were used, and similarly in trials on other drugs or interventions where data on Hb and prevalence of LDA use were reported as baseline characteristics. Moreover, there are also potentially significant diurnal [53] and day-to-day [54] changes in Hb and Hct.

Much used for over a century, in the last twenty years aspirin has been prescribed for many new indications, and at progressively smaller doses. Because of this history, its current use has not been assessed with the rigor of contemporary criteria necessary for licensing a new drug. Perhaps familiarity has bred complacency over associated ADRs. ADRs are difficult to study, are often incompletely reported, and reports of ADRs may not be readily found in databases [55]. It is particularly hard to identify ADRs where the event rate is low and the background rate is not known, and even harder where the ADRs are not clinically obvious. Acute gastrointestinal bleeds severe enough to require hospital admission are, of course, obvious (and relatively easy to count). A gradual fall in Hb or recurrent small bleeds over a long period might not be obvious to the patient or to health professionals, as the effects can be subtle and non-specific (such as increased tiredness) or indirect (for instance worse prognosis in CHF). Development of anemia may well be, by nature, insidious and difficult to identify without serial blood sampling. And LDA might affect Hb other than by causing bleeding from the GI tract (for instance, it has been suggested that LDA might act as an anti-inflammatory drug, tending to increase Hb). Individuals might well differ in their susceptibility to such effects. A clinically significant effect in a small subgroup of patients may not be readily apparent from inspection of mean values for the group as a whole. If, say, LDA caused occult bleeding in a subgroup of 5% of patients such that their Hb fell by 20 g/L and the remaining 95% had no change in Hb, the mean change for the group as a whole would only be 1 g/L.

Individual patient data could be particularly helpful to identify patterns of changes in Hb; some patients might have small amounts of occult gastrointestinal bleeding which occurs from time to time, or they might have persistent bleeding. Sequential measurement of Hb in individuals would be necessary to find out, but appropriate controls would be required as Hb tends to fall in older age [56-59]. (Increasing knowledge of the processes underlying the development of anemia in older people [60,61] may well be of significance for understanding possible effects on Hb due to LDA.) If indeed LDA is significantly associated with falls in Hb, it would be very useful to identify characteristics that make patients potentially more vulnerable - frailty and specific co-morbidities are obvious candidates. The current position of LDA in secondary prevention in cardio- and cerebrovascular disease mean that it is unlikely that in future, trials will be run with LDA compared to placebo, but there might be opportunities in studies in primary prevention (as in the ASPREE trial in progress [28]) or where LDA is used for other conditions.

## Conclusions

It is not clear whether there is a clinically significant effect of LDA on Hb in adults in the absence of overt bleeding, but the limited evidence available at present cannot rule this out and suggests that it may be a problem in older people, or a subset of them. This is an important issue because of the expanding use of LDA by older people and the associations between anemia and impaired function and quality of life, morbidity and mortality.

## Abbreviations

Hb: Hemoglobin; Hct: Hematocrit; WHO: World Health Organization

## Competing interests

The authors declare that they have no competing interests.

## Authors' contributions

HG initiated the study and performed searches and primary data extraction, aided by SD and RAM. All authors read and approved the final manuscript.

## Note added in proof

Since the revised, peer reviewed version of this paper was submitted, another observational study has been published (Al-Azzam SI, AlMahasneh F, Mhaidat N, Alzoubi KH, Khader YS: Prophylactic use of aspirin does not induce anaemia among adults. J Clin Pharm Ther 2010, 35:415-419.) The results of this study are consistent with those of some other observational studies which are included in the review, and do not affect our conclusions.

## Pre-publication history

The pre-publication history for this paper can be accessed here:

http://www.biomedcentral.com/1471-2318/10/71/prepub

## Supplementary Material

Additional file 1 **Specimen search strategy**. One example of search strategies used Click here for file

Additional file 2 **Trial data: LDA as either primary treatment or as comparator drug**. Details of studies and results where LDA has been used in a randomised trial Click here for file

Additional file 3 **ADR data: LDA as either primary treatment or as comparator drug**. Details of studies and results where anemia or change in Hb reported as an ADR Click here for file

Additional file 4 **Prevalence data: LDA use in various settings**. Details of studies and results of prevalence of LDA prescribing and anemia Click here for file
