# Supplementary material for: Is there an association between low dose aspirin and anemia (without overt bleeding)?: narrative review
Source: BMC Geriatr. 2010 Sep 29;10:71. doi: 10.1186/1471-2318-10-71 (PMC2956719; doi:10.1186/1471-2318-10-71)
Supplement: Additional file 1 — Specimen search strategy. One example of search strategies used [file 1471-2318-10-71-S1.PDF]

Additional File 1

Specimen search strategy

PubMed

Search terms : (aspirin OR acetyl salicylic) AND anemia

Field: Title/Abstract

Limits: "Humans" and "All adult: 19+ years" and Publication date 1980 - November 2009.

There were no limits on language or publication type.
