# Supplementary material for: Is there an association between low dose aspirin and anemia (without overt bleeding)?: narrative review
Source: BMC Geriatr. 2010 Sep 29;10:71. doi: 10.1186/1471-2318-10-71 (PMC2956719; doi:10.1186/1471-2318-10-71)
Supplement: Additional file 2 — Trial data: LDA as either primary treatment or as comparator drug. Details of studies and results where LDA has been used in a randomised trial [file 1471-2318-10-71-S2.PDF]

Trial data: LDA as either primary treatment or as comparator drug

Reference	Trial design	Patient details	Daily dose LDA	Duration	Number of patients (observed cases unless stated)		Effect compared with baseline				Comment
		Age, mean ±standard deviation (or range) in years<br><br>Percentage men	mg	Years (unless stated)	Aspirin	Placebo	Outcome	Aspirin*	Placebo*	Difference aspirin vs placebo	
Lewis 1983	RCT DB PC	Mean 56	324	12 weeks	625	641	Number of patients with:				Dispersion in age of
	LDA for prevention of myocardial	All men	effervescent				Fall in Hb >20%	45 (7.2%)	59 (9.2%)	NS	participants NR
	infarction in men with unstable						Fall in Hb 14-20%	136 (22%)	125 (20%)	NS	
	angina						Fall in Hb to <100 g/L	4 (0.6%)	7 (1.1%)	NS	
							FOB >+1	16 (3%)	16 (3%)		
Farrell 1991	RCT DB PC	60±9	300	mean 4	602	594	Fall in Hct, mean±SD %				
	LDA for secondary prevention of	Men 73%	plain or ec				Hct at baseline	44±3.8	44±3.9		
	major cerebro- or cardiovascular						Hct at (mean) 4y	43±4.9	43±5.1		
	disease						Fall from baseline to 4y	0.91±0.20	0.91±0.21	Negligible	
							**Difference vs baseline**	**2P<0.001**	**2P<0.001**		
Meade 1994	RCT DB PC	57 (45-69)	75 cr	2			Rise in Hb				For trial design etc., see
	LDA for primary prevention in	All men			182	187	Hb at baseline	149±14**	151±14**		Meade 1992
	men at high risk of ischemic						Hb at 1y	149±12**	152±11**		Data reported from one
	heart disease						Rise from baseline to 1y	0.4±13**	1.4±11**	NS	subgroup from baseline
							Difference 1y vs baseline	NS	NS		to 1y, and from another
											subgroup from 1y to 2y
					261	263	Hb at 1y	151±13**	151±14**		
							Hb at 2y	151±13**	151±13**		
							Rise from 1y to 2y	0.2±13**	0.4±13**	NS	
							Difference 2y vs 1y	NS	NS		
Diener 1997	RCT DB PC	Mean 67	50	2			Fall in Hb				For trial design etc., see
	LDA for secondary prevention	Men 58%			1211	1219	Mean Hb at baseline	143	144	NR	Bertrand-Hardy 1995
	after transient ischemic attack				1211	1219	Mean Hb at 1y	143	144	NR	and Diener 1996
	or stroke				1211	1219	Mean Hb at 2y	142	143	NR	
							Fall from baseline	NR	NR		Age of patients, 75% ≥ 60 years,
											44% > 70 years
							Fall in Hct				
					1194	1199	Mean Hct at baseline	42.5	42.5	NR	Dispersion in outcome
					1194	1199	Mean Hct at 1y	42.2	42.5	NR	(Hb and Hct) NR
					1194	1199	Mean Hct at 2y	42.3	42.3	NR	
							Fall from baseline	NR	NR		Criteria for "Abnormal
											(low) Hb", "Abnormal
							Number of patients with:				(low) Hct" and "severe
					NR	NR	Abnormal (low) Hb				anemia" NR
					NR	NR	From baseline to 1y	13	11	NR	
							From 1y to 2y	15	15	NR	
					NR	NR	Abnormal (low) Hct				
					NR	NR	From baseline to 1y	9	8	NR	
							From 1y to 2y	10	10	NR	
							Number of patients with:				
							"severe anemia"	1	0		
Hurlen 2006	RCT open study (no placebo	60±8	160 ec	mean 4			Rise in Hb				
	arm)	Men 71%			94		Hb at baseline	133±12			
	LDA for secondary prevention				94		Hb at 3m	142±10			
	of atherothrombotic disease						**Difference vs baseline**	**p<0.001**			
					80		Hb at (mean) 4y	142±12			
							Difference vs baseline	NR			

Silagy 1993	RCT DB PC	73 (70-90)	100 ec	1			Fall in Hb				
	LDA for primary prevention of	Men 49%			200	200	Hb at baseline	142±12	142±11		
	cerebro- or cardiovascular				161	162	**Fall in Hb (mean±SD)**				
	disease						**at 1y**	3.3±8.8	1.1±7.5	**p<0.05**	
							**Difference vs baseline**	**p<0.001**	p=0.06		
							Percentage of patients with				
							**Fall in Hb ≥ 10g /L**	17	9.3	**p<0.05**	
							Fall in Hb≥ 0g /L	63	56	NS	
Nelson 2008	RCT DB PC	76±5	100 ec	1			Fall in Hb				Numbers of patients in
	LDA for primary prevention of	Men 41%			total 209		Hb at baseline	139±13	141±12		each treatment arm NR
	cardiovascular disease				total 192		**Hb at 1y**	137±14	142±13	**P<0.05**	
							**Difference vs baseline**	**p<0.05**	NS		

* Hb and Hct are reported as mean values ± standard deviation, except for ** Meade 1994 where mean values ± standard error are reported

Key: RCT randomized controlled trial DB double blind PC placebo controlled CR controlled release ec enteric coated
m months y years NR not reported NS not significant SD standard deviation SE standard error
