# Supplementary material for: Is there an association between low dose aspirin and anemia (without overt bleeding)?: narrative review
Source: BMC Geriatr. 2010 Sep 29;10:71. doi: 10.1186/1471-2318-10-71 (PMC2956719; doi:10.1186/1471-2318-10-71)
Supplement: Additional file 3 — ADR data: LDA as either primary treatment or as comparator drug. Details of studies and results where anemia or change in Hb reported as an ADR [file 1471-2318-10-71-S3.PDF]

ADR data: LDA as either primary treatment or as comparator drug

Reference	Study design	Patient details	Daily dose LDA	Duration	Number of patients (observed cases unless stated)		ADR			Comment
		Age<br>Mean ±standard deviation (or range) in years<br>Percentage men	mg	Years (unless stated)	Aspirin	Placebo	Outcome	Aspirin	Placebo	
Meister 1984	RCT DB PC<br>Effect of LDA on graft patency and clinical course after aortocoronary bypass surgery	mean 55<br>Men 87%	100	4 m	29	31	Change in Hb or Hct	"Routine laboratory examinations including haemoglobin, erythrocyte count and haematocrit, did not indicate any adverse drug reactions"		No difference between LDA and placebo
Wallentin 1991	RCT DB<br>Effect of LDA on further cardiac ischemic events after unstable coronary artery disease	mean 58 (all <70)<br>All men	75	up to 1	399	397	Change in mean Hb at 1, 3 or 12 m	None	None	No difference between LDA and placebo
Scrutinio 2001	RCT DB<br>LDA as comparator for secondary prevention after acute myocardial infarction	59±10<br>Men 85%	160	6 m	736		Anemia<br>Number of cases of "serious anemia"	1 (0.1%)		
Johnson 2002	RCT open trial<br>LDA as comparator for prevention of graft occlusion and clinical course after peripheral bypass procedures	64±8<br>Men >98%	325	up to 5	413		Anemia<br>Number of cases needing blood tranfusion	4 (1%)		
Matías-Guíu 2003	RCT DB<br>LDA as comparator for secondary prevention of "vascular events" after TIA or CVA	mean 64±10<br>Men 66%	325	mean 2.5	1052		Anemia<br>Number of cases	17 (1.6%)		
Aronow 2008	RCT DB<br>LDA for secondary prevention in atherosclerotic vascular disease; relation between LDA dose and some adverse events	median 63<br>IQR 55-71<br>Men 71%	Lower dose <162<br>(median 100 IQR 81-125)	median 1	2368		Anemia<br>Number of cases (lower dose)	70 (3.0%)		Retrospective subgroup analysis of study of LDA as comparator for secondary prevention of atherosclerotic vascular disease<br>Probable criterion for anemia Hb<90 g/L
		median 62<br>IQR 53-70<br>Men 71%	Higher dose 162-325<br>(median 325 IQR 325-325)		2221		Anemia<br>Number of cases (higher dose)	97 (4.4%)		
Yeomans 2008	RCT DB<br>Esomeprazole vs placebo for reduction of risk of gastroduodenal ulcers (without such ulcers at baseline) in patients all taking LDA	69±7 (all ≥60)<br>Men 57%	75-325<br>(mean±SD 121±57)	26 w	LDA + placebo<br>498		Progressive fall in Hb<br>Number of cases	1 (0.2%)		Reported case was man aged 79y, LDA dose taken NR

Key: RCT randomized controlled trial DB double blind PC placebo controlled
IQR interquartile range SD standard deviation w weeks m months y years
