# Supplementary material for: Is there an association between low dose aspirin and anemia (without overt bleeding)?: narrative review
Source: BMC Geriatr. 2010 Sep 29;10:71. doi: 10.1186/1471-2318-10-71 (PMC2956719; doi:10.1186/1471-2318-10-71)
Supplement: Additional file 4 — Prevalence data: LDA use in various settings. Details of studies and results of prevalence of LDA prescribing and anemia [file 1471-2318-10-71-S4.PDF]

Prevalence data: LDA use in various settings

Reference	Study design	Patient details	Daily dose LDA	Number of patients	Duration						Comment
		Age<br>Mean ±standard deviation (or range) in years	mg		Years (unless stated)	Prevalence of LDA use	Outcome	Aspirin	Placebo/ not taking LDA	Difference aspirin users vs non-users	
	**Community setting**										
Leibovici 1995	Retrospective observational study in a primary care population<br>Prevalence of LDA use for secondary prevention of cardiovascular disease and change in Hb over time (in absence of overt bleeding)	All ages<br>All ages (75% aged ≥60)	250	11500*	mean 25 m<br>range 2-78 m	0.007%<br>Age ≥60 y,<br>M 7.7%<br><br><br><br>F 2.9%	Change in Hb, mean ±SD<br><br>M (n=53)<br>Fall from baseline<br>Difference vs baseline<br><br>F (n=27)<br>Rise from baseline<br>Difference vs baseline	 <br><br>4.7±13<br>p=0.009<br><br><br>0.56±9.3<br>NS			*Population size approximate. Data on change in Hb available from 80/84 pts taking LDA.<br>Additional data; Hb fell by >10% in 12/80 (15%) patients of mean age 69 y
Milman 2004	Observational study of iron status in healthy older people<br>Cohort study with cross-sectional data including prevalence of LDA prescribing and Hb	All aged 80	NR	358		35%	Hb (sic)	NR	NR	NS	
Hammerman-Rozenberg 2006	Observational study<br>Cohort study with cross-sectional data<br>Prevalence of anemia and association with LDA prescribing	All aged 77	100**	464		34%	Hb, mean<br><br>Anemic by WHO criteria	140<br><br>12%	136<br><br>20%	NS<br><br>NS	**dose in "overwhelming majority" of patients taking LDA
	**Studies on cardiovascular disease**										
Sharma 2004	Sub-analysis of data from trial of treatments of chronic heart failure (relationship between Hb and survival)<br>LDA prescription prevalence and mean Hb reported	72±7	Probably LDA	3044		59%	Hb, mean	139	141	p=0.01	Retrospective analysis
Portnay 2005	Cohort study of (chronic) prior aspirin use and outcome after admission to hospital with acute myocardial infarction	≥65	Probably 81-325 for most patients	118,992		33%	Hb, mean ±SD	136±18	135±18	p<0.0001	
Muzzarelli 2006	Sub-analysis of data from trial of treatments of chronic angina (relationship between anemia and prognosis)<br>LDA prescription prevalence and anemia reported	≥75	Probably LDA	253		85%	Numbers of patients anemic (WHO criteria)	62 (29%)	11 (29%)	No difference	Retrospective analysis

	**Investigation of iron deficiency anemia (IDA)**					
Black 1999	Prevalence of regular aspirin prescribing in a primary care population of older patients.	>74	NR Probably LDA	6288	11%	
	Audit of sub group referred to secondary care for investigation of IDA	mean 81		112	24%	
	Audit of secondary subgroup in which no cause of IDA was found	NR		59	41%	
Luman 2003	Audit of investigation of patients referred to secondary care for investigation of IDA but with no cause found	Men all ages Women >45	NR LDA	141	25%	
Niv 2005	Audit of patients referred to secondary care for investigation of IDA without gastro-intestinal symptoms	Men >50 Women post-menopausal	100 or 325 Presumed to be once daily dose	48	29%	
Ioannou 2007	Prospective study of investigation of patients found to be anemic when admitted as inpatients to general medical services (excluding acute GI bleeds)	62±17	NR Probably LDA	637***	37%	*** Anemic by WHO criteria (mean Hb 104±17 g/L)

Key: M men F women m months y years NR not reported NS not significant SD standard deviation
